# Advances in studies of tyrosine kinase inhibitors and their acquired resistance

**DOI:** 10.1186/s12943-018-0801-5

**Published:** 2018-02-19

**Authors:** Qinlian Jiao, Lei Bi, Yidan Ren, Shuliang Song, Qin Wang, Yun-shan Wang

**Affiliations:** 1 International Biotechnology R&D Center, Shandong University School of Ocean, 180 Wenhua Xi Road, Weihai, Shandong 264209 China; 2 School of Preclinical Medicine, Nanjing University of Chinese Medicine, 138 Xianlin Road, Nanjing, Jiangsu 210023 China; 3 Department of Anesthesiology, Qilu Hospital, Shandong University, 107 Wenhua Xi Road, Jinan, 250012 China

**Keywords:** Cancer, Protein tyrosine kinase, Tyrosine kinase inhibitors, Acquired resistance

## Abstract

Protein tyrosine kinase (PTK) is one of the major signaling enzymes in the process of cell signal transduction, which catalyzes the transfer of ATP-γ-phosphate to the tyrosine residues of the substrate protein, making it phosphorylation, regulating cell growth, differentiation, death and a series of physiological and biochemical processes. Abnormal expression of PTK usually leads to cell proliferation disorders, and is closely related to tumor invasion, metastasis and tumor angiogenesis. At present, a variety of PTKs have been used as targets in the screening of anti-tumor drugs. Tyrosine kinase inhibitors (TKIs) compete with ATP for the ATP binding site of PTK and reduce tyrosine kinase phosphorylation, thereby inhibiting cancer cell proliferation. TKI has made great progress in the treatment of cancer, but the attendant acquired acquired resistance is still inevitable, restricting the treatment of cancer. In this paper, we summarize the role of PTK in cancer, TKI treatment of tumor pathways and TKI acquired resistance mechanisms, which provide some reference for further research on TKI treatment of tumors.

## Background

Malignant tumors have always been a serious threat to human life. Although the diagnostic and therapeutic levels have improved, many kinds of tumor survival rates have remained low. Anti-tumor research remains a challenging and significant field in the life sciences today. At present, the commonly used anti-tumor drugs are cytotoxic drugs. Cytotoxic cancer drugs are usually of high acute toxicity, which have the disadvantages of poor selectivity, strong side effects and easy to produce drug resistance [1]. In recent years, with the rapid progress of life science research, signal transduction in tumor cells, cell cycle regulation, induction of apoptosis, angiogenesis, the interaction of cells and extracellular matrix and other basic processes are being gradually clarified [2]. In addition, it is pointed out that these drugs may be very specific to certain cellular targets (e.g. DNA, tubulin) present in cancer as well as in normal cells. Using the key enzymes of cell signal transduction pathway associated with tumor cell differentiation and proliferation as drug screening targets, and developing high efficiency, low toxicity and specificity of new anticancer drugs acting on specific targets have become important direction of research and development of antitumor drugs today [3].

Protein tyrosine kinase (PTK) is a class of proteins with tyrosine kinase activity that catalyzes the transfer of phosphate groups on ATP to the tyrosine residues of many important proteins, making proteins phosphorylation, then transferring signal to regulate cell growth, differentiation, death and a series of physiological and biochemical processes [4]. PTK disorders can cause a series of diseases in the body. Previous studies have shown that more than 50% of the proto-oncogene and oncogene products have PTK activities, their abnormal expression will lead to cell proliferation regulation disorders, causing tumorigenesis finally [5]. In addition, PTK abnormal expression is also associated with tumor invasion and metastasis, tumor neovascularization and tumor chemotherapy resistance [6]. Therefore, PTK as a target for drug research and development has become a hot spot for anti-tumor drug research.

By the end of the century, so-called targeted cancer therapy with reduced side effects was made possible by advances such as specific monoclonal antibodies that bound to unique epitopes on the surface of cancerous cells and by small molecules. International major research institutions, pharmaceutical groups have attached great importance to PTK as the target drug research, such as selective tyrosine kinase inhibitors (TKI) that affected specific molecular pathways up-regulated in certain cancers [1]. The pro-spective molecular profiling of cancers to find such ‘driver’ abnormalities became feasible in clinical practice, allowing for routine genotype-directed rather than empiric therapy. In 2001, the first TKI drug imatinib was quickly approved by the FDA and opened up new ideas for cancer treatment. Until 2018 or beyond, a total of more than 20 kinds of TKI approved by the FDA [7–13], drug-related information as listed in Table 1. These drugs have high selectivity, high efficacy, low side effects, ease of preparation, and have superiority in the treatment of chronic myeloid leukemia(CML), non-small cell lung cancer(NSCLC), renal cell carcinoma(RCC) than traditional cytotoxic antineoplastic agents [14], some have become the first-line drug for the treatment of cancer.

**Table 1:** TKI launched on market

TKI	Time to market	Development company	Target	Application of disease
Imatinib	2001	Novartis	Abl, PDGFR, SCFR	CML, GIST
Gefitinib	2003	AstraZeneca	EGFR	NSCLC
Nilotinib	2004	Novartis	Bcr-Abl, PDGFR	CML
Sorafenib	2005	Bayer	Raf, VEGFR, PDGER	Advanced RCC
Sunitinib	2006	Pfizer	PDGFR, VEGFR,	GIST, Advanced RCC
Dasatinib	2006	Bristol-Myers Squibb	Bcr-Abl, SRC, PDGFR	CML
Lapatinib	2007	GlaxoSmithKline	EGFR	Breast cancer
Pazopanib	2009	GlaxoSmithKline	VEGFR, PDGFR, FGFR	Advanced RCC,STS,NSCLC
Crizotinib	2011	Pfizer	ALK	NSCLC
Ruxolitinib	2011	Novartis	JAK1, JAK2	myelofibrosis
vandetanib	2011	AstraZeneca	VEGFR, EGFR	Advanced Thyroid cancer
Axitinib	2012	Pfizer	VEGFR	Advanced RCC
Bosutinib	2012	Wyeth	Abl, SRC	CML
Afatinib	2013	Boehringer Ingelheim	EGFR	NSCLC
Erlotinib	2013	Roche	EGFR	NSCLC
Ceritinib	2014	Novartis	ALK	NSCLC
Osimertinib	2015	AstraZeneca	EGFR	NSCLC
Lenvatinib	2015	Eisai	VEGFR	DTC
Alectinib	2015	Roche	ALK	NSCLC
Regorafenib	2017	Bayer	VEGFR, EGFR	HCC, CRC,GIST
Neratinib	2017	Puma	HER2	Breast cancer
Brigatinib	2017	Ariad	ALK	NSCLC

Although TKI has made great strides in the treatment of cancer, it still faces some challenges. Because even in highly sensitive patients with TKI, tumor cells can always be self-adjusting, looking for a way out, to avoid TKI target, acquired resistance and the progress of the disease is still inevitable [15]. The median effective time for TKI therapy was only 5 to 9 months [16]. With our increased understanding of the spectrum of acquired resistance to TKI, major changes in how we conduct clinical research in this setting are now underway. In order to fight against resistance to TKI, the investigators should further study the mechanisms of their resistance and suggest a regimen that prevents or treats their resistance.

## PTK and tumor

PTK is only found in multicellular animals and is an enzyme that activates and regulates cell proliferation signaling pathways. According to its structure, it can be divided into two categories: Receptor PTK (RTK) and Non-receptor PTK (NRTK). These two types of PTK can be further divided according to their structural homology multiple enzymes. Analysis of human genome data shows that there are 518 kinase genes in the human body, of which 90 have been identified PTK, including RTK 58 species and NRTK 32 species [17].

**Fig. 1:**
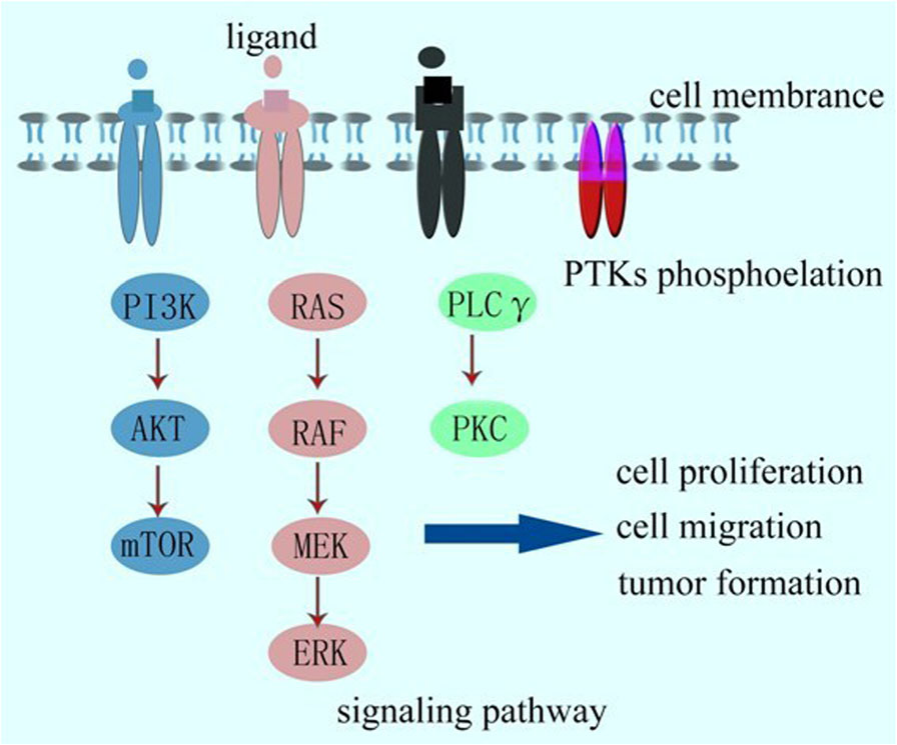
Cell signaling pathways induced by RTK. RTK can bind to ligands and phosphorylate tyrosine residues of target proteins and transmit information through PI3K/AKT/mTOR; RAS/RAF/MEK/ERK; PLCγ/PKC and other signaling pathways to activate a series of biochemical reactions; or different information combined to cause a comprehensive cellular response, including cell proliferation, cell migration and tumor formation

RTK includes epidermal growth factor receptor (EGFR), platelet-derived growth factor receptor (PDGFR), vascular endothelial growth factor receptor (VEGFR) and insulin receptor (InsR) family and so on. They usually have an extracellular domain that binds to a specific ligand, a transmembrane region, and an intracellular kinase domain that selectively binds to and phosphorylates the substrate [18]. RTK can bind to ligands and phosphorylate tyrosine residues of target proteins and transmit information through PI3K/AKT/mTOR; RAS/RAF/MEK/ERK; PLCγ/PKC and other signaling pathways to activate a series of biochemical reactions; or different information combined to cause a comprehensive cellular response (such as cell proliferation) (Fig. 1) [19]. Clinical studies in cancer have shown that these receptors and their ligands are important in many tumors, and many cancers have over-expressed growth factors that cause excessive tyrosine phosphorylation signal into cells [20].

NRTKs generally have no extracellular structure. They are usually coupled to the cell membrane or present in the cytoplasm, including Abl kinase, Src kinase family and so on [21, 22]. NRTK performs signal transduction primarily through cytokine receptors, T-cell receptors and other signaling pathways. T lymphocyte receptors, B lymphocyte receptors, immunoglobulin receptors and so on can recruit NRTK, and then through tyrosine phosphorylation to form signal transduction complex, and then activate the downstream signal transduction, promote cells proliferation, lead to the formation of tumors [23].

Overexpression of the PTK gene enhances the activity of PTK and changes its downstream signaling pathways, causing cell proliferation disorders and eventually leading to tumor formation [5]; mutations in tumor tissue may cause PTK to spontaneously activate in the absence of a stimulus source or appear abnormal growth rate [24]; clinical studies have shown that PTK overexpression or decreased expression can show the biological characteristics of the tumor or predict the response to treatment and survival [25].

### EGFR family

The human EGFR gene is localized to the short arm of chromosome 7 (7p12.3-pl2.1), which encodes a product consisting of 1210 amino acids with a molecular weight of about 170 kb. EGFR is a cell surface receptor and plays a pivotal role in regulating survival and apoptosis of epithelial cells and tumors of epithelial cell origin. Overexpression of EGFR and its ligands is present in a variety of epithelial tumor cells such as lung cancer, breast cancer, bladder cancer, prostate cancer and squamous cell carcinoma of the head and neck [26–29]. It is a member of the ErbB family, a group of four receptor tyrosine kinases sharing similarities in structures and functions: ErbB1 (EGFR or HER1), ErbB2 (HER2), ErbB3 (HER3) andErbB4 (HER4). In breast cancer, overexpression of HER2 is found in approximately 10%–30% of patients and is associated with reduced survival [30]. In addition, EGFR deletion can also be detected in malignant gliomas, NSCLC, breast cancer, medulloblastoma and ovarian cancer [31, 32]. The most common EGFR deletion mutant is EGFR VIII. EGFR VIII lose ligand binding region, but can activate tyrosine kinase itself, stimulate the activation of downstream signaling pathways, and not dependent on its ligand binding region [33]. EGFR overexpression and/or mutation through signal transduction lead to cell growth out of control and malignancy in many tumors. In patients with high expression of EGFR, the degree of malignancy is high, the recurrence interval is short, the recurrence rate is high, the survival time of the patients is short [34].

### VEGFR family

VEGFR family members include VEGFR1, VEGFR2 and VEGFR3. The family of receptors has 7 immunoglobulin-like domains in the extracellular domain and a hydrophilic insert sequence in the intracellular tyrosine kinase region [35]. In the malignant growth and metastasis of solid tumors, neovascularization of the tumor plays a very important role, providing the necessary nutrients and oxygen for the tumor growth [36]. VEGF plays an important role in the proliferation, migration, and vascularization of endothelial cells as the most powerful vascular penetrant and endothelium-specific mitotic source [37]. There was a significant positive correlation between the VEGFR expression level and the degree of vascularization and malignancy of tumor tissue.

VEGF is mainly acting on high affinity of the recipient VEGFR1 and VEGFR2 in the vascular endothelial cells and play its biological role, both have different signal transduction pathways [38, 39]. Among them, VEGFR2 is the most important in mediating the biological effect of VEGF, which is closely related to cell chemotaxis, cell division and act in recombination [40]. VEGFR1 has stronger affinity binding to VEGF, and phosphorylation is similar, but the effect of cell division is much smaller [41]. VEGFR3 is highly expressed in the blood vessels of the embryonic vessels, veins and lymphatic vessels, but after the development of the fetus, VEGFR3 only in the lymphoid endothelial cells. In a variety of tumor course, VEGFR3 induced tumor lymph angiogenesis, promoting tumor lymph node invasion and lymph node metastasis. VEGFR3 plays an important role in aiding cellular viability and blocking VEGFR3 signaling hinders this ability, which may induce autophagy [42, 43].

### PDGFR family

In addition to PDGFRα and PDGFRβ, members of the PDGFR family also include the colonial stimulating factor-1 receptor (CSF1R), the stem cell growth factor receptor (SCGFR), FLK2/FLK3. The family of receptors has 5 immunoglobulin-like domains in the extracellular domain and a hydrophilic insert sequence in the intracellular tyrosine kinase region [44]. PDGFR is mainly present in fibroblasts, smooth muscle cells, but also expression in the kidney, testis and brain. PDGFR is closely related to tumorigenesis [45]. In most glioblastomas, autocrine loop of PDGF and its receptors is formed. This loop is closely related to the occurrence and development of tumor [46]. In addition, similar loops are also present in melanoma, meningiomas, neuroendocrine tumors, ovarian cancer, prostate cancer, lung cancer and pancreatic cancer [47, 48].

### InsR family

InsR family members include INSR, IGF1R and IRR three members. IGF-I and IGF-II have the effect of promoting proliferation and inhibiting apoptosis in breast cancer, cervical cancer, colon cancer and lung cancer [49–51]. IGF1R is overexpressed in breast cancer, cervical cancer, and have a great impact on the pathological process of breast cancer [52]. In addition, IGF1R is associated with the metastasis of melanoma at the end of the eye pigment, which is a predictor of this type of tumor metastasis.

### Src family

**Fig. 2:**
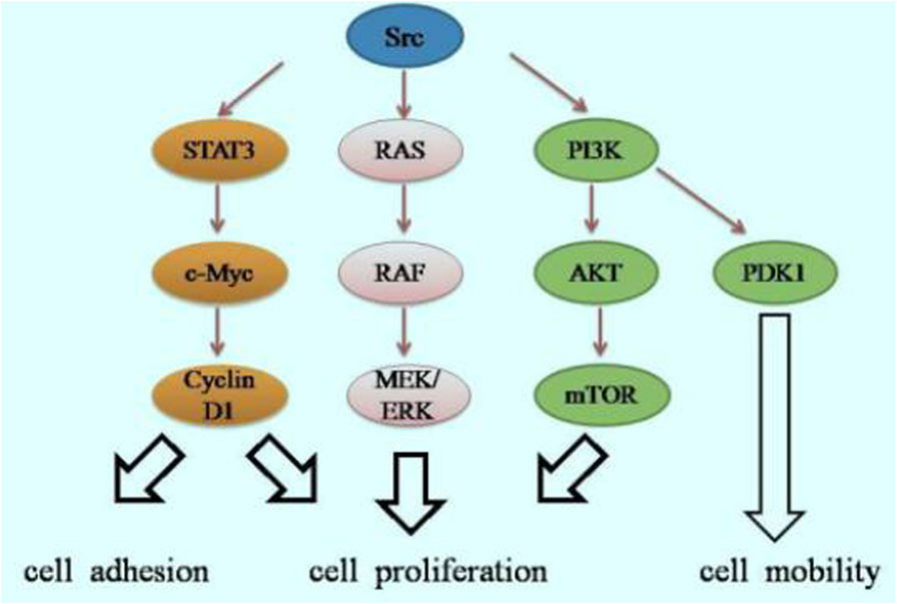
Cell signaling pathways induced by Src kinases. Src kinases regulate a broad spectrum of cellular events such as cell adhesion, proliferation and mobility. These include the STAT3 pathway that regulates the expression of c-Myc and Cyclin D1; the RAS/RAF/MEK/ERK pathway; and the PI3K/AKT/mTOR pathway

Src is an important member of NRTK, which plays a key role in the regulation of many cells through the extracellular ligand binding to the receptor and the cell adhesion molecule activationin cell cycle specific stage [53–56]. These include the RAS/RAF/MEK/ERK pathways; the PI3K/AKT/mTOR pathway; and the STAT3 pathway that regulates the expression of c-Myc and Cyclin D1 (Fig. 2).It can affect cell adhesion, mobility, proliferation and angiogenesis. Under normal circumstances, the activity site of Src kinase closed, its expression was inhibited. But under the action of exogenous or endogenous carcinogenic factors, kinase hyperactivated, cell proliferation and differentiation become uncontrolled and lead to tumorigenesis [56, 57].

### Abl family

**Fig. 3:**
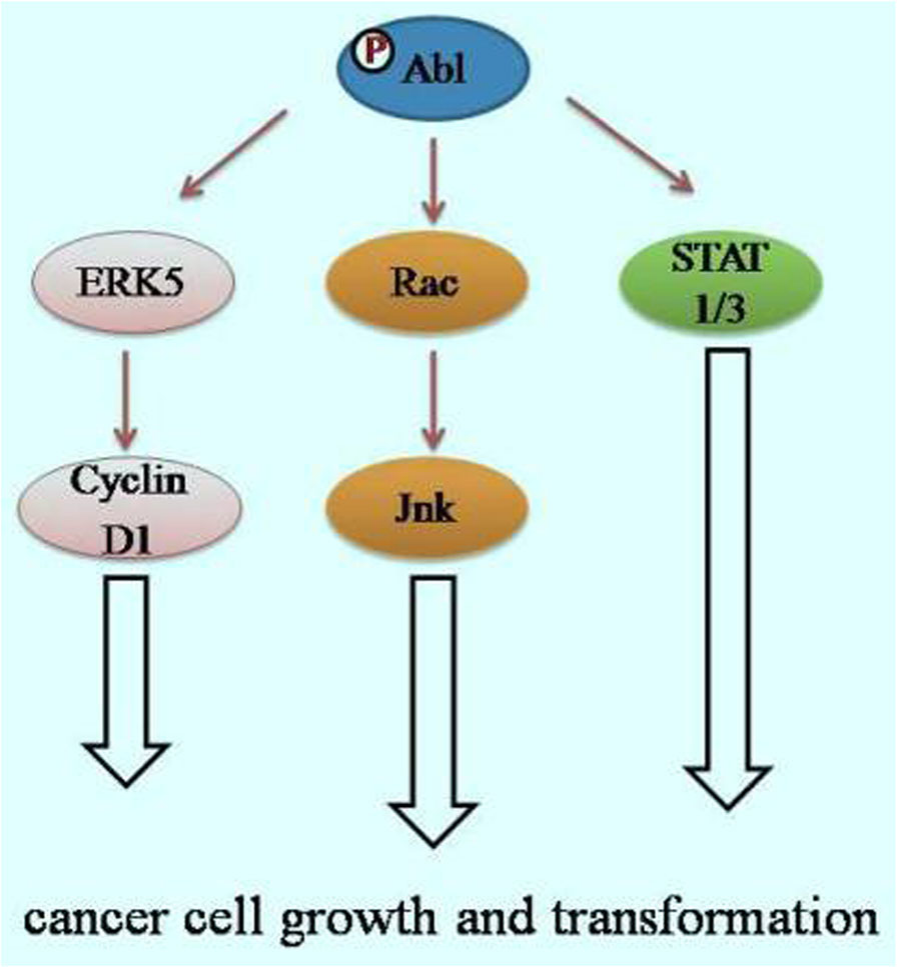
Cell signaling pathways induced by Abl kinases. Phosporylated Abl activates oncogenic signaling pathways by activation of ERK5; Rac/Jnk, and STAT 1/3 pathways. These cascades are required for cancer cell growth and transformation

Abl family includes two members: c-Abl and Arg. Both proteins can be localized in cytosol, cell membranes, and the actin cytoskeleton. Additionally, c-Abl is also present in the nucleus [58]. In normal cells, c-Abl contributes to actin remodeling, cell adhesion and motility, DNA damage response, and microbial pathogen response. Deregulation and aberrant expression of c-Abl kinases has been implicated in several types of cancer, such as breast cancer [59, 60], colon cancer [61], and NSCLC [62]. Phosporylated c-Abl activates oncogenic signaling pathways by activation of ERK5, Rac/Jnk, and STAT 1/3 pathways (Fig. 3). c-Abl is also known to be important for the genesis of CML, where it forms the oncogenic fusion protein with Bcr after the translocation of a part of chromosome 9 to chromosome 22 [63].

## TKIs biology

TKI can compete ATP binding site of tyrosine kinase with ATP, reduce tyrosine kinase phosphorylation, thereby inhibiting cancer cell proliferation. It has the characteristics of high selectivity, small adverse reaction and convenient oral administration [64]. According to the main targets of different, these TKI can be divided into EGFR inhibitors, VEGFR inhibitors, anaplastic lymphoma kinase (ALK) inhibitors and Bcr-Abl inhibitors. The anti-tumor mechanism of TKI can be achieved by inhibiting the repair of tumor cells, blocking the cell division in G1 phase, inducing and maintaining apoptosis, anti-angiogenesis and so on [65–67].

### EGFR inhibitors

Gefitinib is a selective EGFR-TKI, which is usually expressed in epithelial-derived solid tumors. Inhibition of EGFR tyrosine kinase activity can prevent tumor growth, metastasis and angiogenesis, and increase tumor cell apoptosis [68, 69]. In vivo, gefitinib extensively inhibited tumor growth of human tumor cell derived lines in nude mice and increased the antitumor activity of chemotherapy, radiotherapy and hormone therapy. It has been shown in clinical trials that gefitinib has antitumor responses to locally advanced or metastatic NSCLC and can improve disease-related symptoms [8, 70].

Lapatinib is a reversible dual inhibitor of EGFR and HER2. Lapatinib can inhibit both EGFR and HER2 tyrosine kinases [12, 71, 72]. Lapatinib inhibits MAPK and PI3K signal transduction in EGFR and HER2 overexpressing tumor cell lines [73, 74]. The response to lapatinib was significantly associated with HER2 overexpression, which inhibited phosphorylation of HER2, RAF, AKT and ERK. Lapatinib has been approved by the FDA in 2007 for the treatment of breast cancer, NSCLC, head and neck cancer and gastric cancer [75].

Erlotinib can inhibit the phosphorylation of intracellular tyrosine kinases associated with EGFR, causing cell growth arrest and/or cell death. This medicine is used for third-line treatment of locally advanced or metastatic NSCLC after a previous failure of at least one chemotherapy regimen [76], combined with gemcitabine for first-line treatment of locally advanced unresectable or metastatic pancreatic cancer [77, 78]. Erlotinib treatment window is very narrow, the recommended dose close to the maximum tolerated dose, more than the recommended dosage may occur unacceptable serious adverse reactions, such as breathing difficulties, cough, diarrhea, rash and so on [79].

### VEGFR inhibitors

Sorafenib can inhibit RAF-1, VGFR-2 and VGFR-3 and other RTK activity [80]. It is the first anti-tumor drugs targeting and inhibiting RAF kinase and VEGFR kinase at the same time [81, 82]. It can directly inhibit the proliferation of tumor cells by blocking the cell signaling pathway mediated by RAF/MEK/ERK [83], but also through the action of VEGFR to inhibit the formation of angiogenesis and cut off the nutritional supply of tumor cells to limit the tumor growth [84, 85]. The clinical studies have shown that sorafenib can significantly prolong the progression-free survival of patients with kidney cancer, its major adverse reactions are nausea, diarrhea, rash and high blood pressure [86].

Sunitinib is a multi-target kinase inhibitor that targets VEGFR, PDGFR-α, PDGFR-β, CSF-1R, and the like. It is used to treat inoperable RCC [87] and imatinib-resistant or intolerant gastrointestinal stromal tumors (GIST) [88]. The drug is well tolerated in children with recurrent/refractory gliomas or ependymomas, but specific monotherapy options need further investigation and may be considered in combination with radiotherapy and/or chemotherapy [89].

### ALK inhibitor

ALK belongs to the insulin receptor superfamily. The physiological function of ALK in the normal body is not clear, the research suggests that it has a role for nervous system function [90, 91]. Crizotinib is a multi-target tyrosine kinase receptor inhibitor directed against ALK and acts on hepatocyte growth factor receptor (HGFR) in addition to ALK for the treatment of ALK-positive patients with locally advanced or metastatic NSCLC [92]. The study found that crizotinib also has a good anti-tumor effect on patients with NSCLC rearranged gene encoding proto-oncogene receptor (ROS1), the FDA approved in March 2016 its scope of application to broaden to ROS1-positive NSCLC patients [93].

Ceritinib is an oral small-molecule tyrosine kinase inhibitor targeting ALK, IGF-1R, InsR and ROS1, with a highly selective effect on ALK [94]. The main mechanism of action of ceritinib is to inhibit the phosphorylation of ALK itself and ALK-mediated downstream signal proteins, thereby inhibiting the proliferation of ALK-positive cancer cells. It is clinically used to treat ALK positive metastatic NSCLC or NSCLC that is exacerbated and intolerable to be treated with crizotinib [95].

### Bcr-Abl kinase inhibitors

Imatinib has three main targets: various Abl, SCGFR and PDGFR, the effect is to inhibit the target-mediated cell events [96]. The molecular mechanism of imatinib is as an ATP inhibitory inhibitor, blocking PTK phosphorylation, inhibiting Bcr-Abl expression, thereby preventing cell proliferation and tumor formation [97, 98]. However, Bcr-Abl products have multiple effect, a single pathway of inhibition cannot completely eliminate the malignant proliferation of tumor cells, so this product is only efficient rather than special effects of anti-cancer drugs [99].

Bosutinib is a dual inhibitor of Abl and Src kinases [100, 101]. Bosutinib has a high anti-proliferative activity, can inhibit the proliferation and survival of CML cells [102]. It can inhibit the activity of CML graft in vivo, making K562 tumor transplant cells subsided in nude mice. The inhibitory activity to Abl kinase is considered to be the main reason for against the proliferation of chronic myeloid leukemia cells [103, 104].

## TKI acquired resistance

Most cancer patients can relieve disease after using TKI, but acquired resistance remains a bottleneck in cancer targeted therapy [105]. TKI has a variety of mechanisms for drug resistance, the current researchers in the acquired resistance mechanism and its treatment strategy research has made great progress.

### T790M mutation

T790M mutation is the first recognized acquired resistance mechanism after the TKI treatment. T790M mutation is due to EGFR gene 20 exon 790th codon missense mutation, resulting in the product from threonine to methionine [106]. 43%-50% of patients with NSCLC who were resistant to gefitinib or erlotinib were positive for T790M mutations [107]. The cause of resistance may be methionine instead of threonine, a steric hindrance, which affects the formation of hydrogen bonds between tyrosine kinases and TKI, leading to the inability of TKI to bind [108, 109]; Other studies have shown that T790M mutation and EGFR-sensitive mutations results in increased intracellular ATP affinity, whereas the affinity for TKI is reduced, resulting in TKI acquired resistance [110].

More and more studies further support the T790M mutation is an important acquired resistance mechanism in TKI therapy. T790M mutation will increase the tyrosine kinase activity, enhance tumorigenicity [111], but this type of drug-resistant patients still shows the slow progress of the disease trend. After immediate withdrawal, the disease has the possibility of outbreak, and targeted therapy is still effective after treatment interruption, which may be due to drug-resistant tumor cells still exist in a certain proportion of cells sensitive to EGFR-TKI, but the specific mechanism is not clear [112].

In recent years, EGFR irreversible inhibitors have given new hope to patients with failed EGFR-TKI therapy. These drugs act on the ATP binding site of EGFR, covalently bind to the receptor kinase region, and simultaneously inhibit multiple members of the EGFR receptor family [113]. Therefore, theoretically, it can still play an inhibitory effect to the second mutation of T790M EGFR, increase the efficacy and reduce the occurrence of drug resistance [114].

Aftinib (BIBW2992) is a new generation of representative irreversible potent oral inhibitors that simultaneously inhibit EGFR and HER2 targets [115]. BIBW2992 further delays tumor progression by maintaining irreversible binding to EGFR and HER2, maintaining longer activity, suppressing transformation in isogenic cell-based assays, inhibits survival of cancer cell lines and induces tumor regression in xenograft and transgenic lung cancer models, with superior activity over erlotinib [115, 116]. BIBW2992 can benefit clinical patients with refractory NSCLC.

Dacomitinib (PF299) is an oral small molecule that irreversibly inhibits EGFR, HER2 and HER4 tyrosine kinase inhibitors. In vivo and in vitro experiments, it showed potency of T790M mutations and EGFR20 exon insertion mutations, which could overcome EGFR-TKI acquired resistance by inhibition of T790M mutations.

Third generation EGFR TKIs are designed to target EGFR TKI sensitizing mutations and the T790M resistance mutation, thus inhibiting the growth of EGFR T790M-positive tumors. By sparing wild-type EGFR, these compounds are also anticipated to reduce the toxicities that have been associated with first- (e.g. gefitinib; erlotinib) and second-generation (e.g. afatinib) EGFR TKIs. Osimertinib (AZD9291, Tagrisso™), an orally administered, third generation EGFR TKI, has been approved in numerous countries for using in patients with T790M-positive advanced NSCLC [117]. Osimertinib was approved by the FDA, whereas ASP8273 is currently in clinical trials to evaluate the efficacy in patients with T790M-positive EGFR-mutated NSCLC [118].

### c-MET gene amplification

Human c-MET gene located in chromosome 7, the coding product is a specific receptor for hepatocyte growth factor. MET occurred amplification, mutations and overexpression in a variety of tumors [119, 120]. After combined with HGF, MET can activate RTK system, promoting cell proliferation and differentiation, inducing epithelial cell migration and inducing angiogenesis. 20% of NSCLC patients with TKI resistance are related to c-MET gene amplification, but its occurrence was not related to the presence of T790M mutation [121]. In the presence of EGFR-TKI, c-MET gene amplification activates ERBB3-PI3K signaling pathway, directly activating EGFR downstream signaling pathway, leading to NSCLC resistance to TKI. Studies have shown that MET may be treatment targets after TKI acquired drug [122].

With the discovery of c-MET gene amplification mechanism, the combination of TKI has become another new idea to overcome the resistance of EGFR-TKI [123, 124]. MetMAb is a unique single-arm antibody that blocks the MET receptor. It inhibits the binding of HGF to the MET receptor and restores its sensitivity to erlotinib [125].

ARQ197 is a novel selective TKI that stabilizes the non-activated conformations of c-MET1 by non-ATP competitive inhibition and inactivates c-MET [126]. In vivo antitumor activity, the antitumor activity of ARQ197 combined with EGFR-TKI was found to be greater than that of ARQ197 and EGFR-TKI mono therapy. At present, ARQ-197 and erlotinib were combined to therapy advanced or metastatic non-small cell lung cancer in the three stages of research [127, 128].

### Loss of PTEN expression

The PTEN gene is another tumor suppressor gene that is closely related to tumorigenesis and progression [129]. In the study of PC-9 cell lines resistant to gefitinib, p-AKT in the cell line was significantly up-regulated and the expression of PTEN was reduced. Thus, the expression of PTEN was absent and the tumor cells could find independent on EGFR activation pathway, but effectively activate the PI3K pathway, resulting in resistance to EGFR-TKI treatment [130]. Immunohistochemical staining revealed that 93 NSCLC patients treated with gefitinib had 19 deficient PTEN expression, but this had nothing to do with the objective response rate, the progression of disease, and the overall survival time. This also indicates that EGFR-TKI resistance resulting from loss of PTEN expression is associated with structural changes in EGFR downstream signaling [131].

### IGF-1R-mediated EGFR downstream pathway activation

The IGF-1R is overexpressed in many tumors, making the proto-oncogene transcription and translation, and promoting tumor cell growth [132]. IGF-1R activates both RAS/RAF/MAPK and PI3K signaling pathways [133]. In the study of cell lines, IGF-1R leads to EGFR-TKI resistance by regulating the metabolism, proliferation and apoptosis of tumor cells and continuously activating the PI3K-AKT signaling pathway. Studies have found that inhibition of IGF-1R-mediated activation of EGFR downstream pathway can prevent or delay the emergence of drug-resistant after receiving Gefitinib treatment [134, 135].

EGFR pathway downstream signaling molecule PIK3A mutation or/and amplification make ERBB3-mediated PI3K signal transduction pathway activation, PTEN gene deletion or/and mutation can lead to AKT signal activation [136]. Finally, they make resistance to EGFR-TKI. BKM120 is an oral PI3K inhibitor. Preclinical studies have shown it has antitumor activity on malignant tumor with PTEN mutation or/and deletion or PI3K mutation or/and amplification [137, 138].

AKT pathway activation is commonly associated with acquired resistance to EGFR-TKI treatment in NSCLC harboring a diverse array of other, previously identified resistance mechanisms. AKT activation is a convergent feature of acquired EGFR tyrosine kinase inhibitor resistance, across a spectrum of diverse, established upstream resistance mechanisms. Studies have shown that AKT inhibition, specifically, could moreuniformly enhance response and survival in patients with high pAKT levels who are at high risk for AKT-mediated resistance, as this distinct approach has the unique potential to combat the otherwise profound heterogeneity of molecular resistance events that are present in EGFR-mutant NSCLC patients with acquired EGFR-TKI resistance to improve their outcomes [139, 140].

### EML4-ALK fusion gene

Echinoderm microtubule associated protein-like 4-anaplastic lymphoma kinase (EML4-ALK) fusion gene is lung cancer-driven gene [141], EML4 and ALK two genes located on human chromosome 2 p21 and p23, intracellular ALK gene and with the N-terminal EML4 inverted fusion induces tyrosine kinase activity by stimulating the PI3K/AKT/MAPK signaling pathway, resulting in the proliferation and differentiation of tumor cells and the inhibition of apoptosis [142]. EML4-ALK fusion gene accounts for 3% to 7% of NSCLC, mostly in non-smoking, young female patients with adenocarcinoma [143]. For the EML4-ALK fusion gene, many highly effective ALK-TKIs have emerged clinically, including the second generation of ceritinib, Brigatinib and the third generation of Loratinib.

### Amplification of ALK fusion gene copy number

Amplification of ALK fusion gene copy number is one of the possible mechanisms of crizotinib resistance. In one study, extensive amplification of the ALK fusion gene was detected in 18 patients with lung adenocarcinoma resistant to crizotinib and in acquired drug resistant cell lines with H3122 (including EML4-ALK mutant 1) [144]. ALK signaling pathway is often retained when the ALK fusion gene has a second mutation or increased copy number in the kinase domain and plays a role in tumor survival and drug resistance. Therefore, the use of more effective second- and third-generation ALK inhibitors may be able to overcome the secondary resistance problems caused by these mechanisms.

### Activation of signal bypass

ALK belongs to the tyrosine kinase, and its downstream signaling pathways mainly include PI3K/AKT/mTOR, RAS/MEK/ERK and JAK3/STAT3, these signals are related to cell survival and proliferation, crizotinib through its specificity apoptosis was induced by inhibiting the expression of downstream signal of EML4-ALK [145]. When the signal is activated bypasses, the signal transduces around the original target of the inhibitor and activates downstream signals through the signal bypasses, leaving crizotinib not sufficient to suppress tumor growth, leading to drug resistance. These ALK-independent mechanisms of resistance include activation of EGFR, KIT, IGF-1R and other signaling pathways.

### Epithelial mesenchymal transformation

The epithelial mesenchymal transformation (EMT) refers to the transformation of epithelial cells into interstitial cells. Through EMT, the epithelial cells lose the polarity of the cells, lose the epithelial phenotype such as the connection with the basement membrane, obtain higher interstitial phenotypes such as migration and invasion, anti-apoptosis and degradation of the extracellular matrix capacity [146, 147]. EMT is an important biological process in which epithelial cell-derived malignant cells acquire the ability to migrate and invade. In recent years, a number of studies have shown EMT is related to tumor stem cell formation, drug resistance and tumor metastasis.

### Other possible resistance mechanisms

BRAF gene encoding BRAF protein is the molecular isomer of RAF protein, locates downstream of EGFR signaling pathway. It activated MAPK, promoted cell proliferation and differentiation through the interaction with RAS [148–150]. There were about 3% of BRSC gene mutations in NSCLC patients. It has been reported that BRAF gene mutation is one of the resistance mechanisms of EGFR monoclonal antibody in the treatment of colorectal cancer [151]. It has also been reported that mTOR is associated with EGFR resistance, blocking mTOR pathway can interfere with tumor growth [152]. In addition, TKI acquired resistance is also associated with increased VEGF levels, and VEGFR/EGFR dual pathway inhibitors have been shown to have a good therapeutic effect in EGFR-TKI-resistant patients [153].

## Conclusions

The mechanism of tumor drug resistance is complicated, and the new emerging mechanism remains to be further studied. On one hand, tumor has a multi-drug resistance mechanism or a escape pathway, combined treatment is possible to block the signal path. In clinical practice, we often need combined application of a number of different drugs to affect the tumor growth [154]. On the other hand, gene therapy technology can inhibit the expression of drug resistance gene mRNA, with a broad clinical application prospects [155, 156]. As the researchers on the tumor resistance mechanism continuously deepening and related treatment technology continuously develop, human can improve the effect of clinical chemotherapy, reverse the resistance of cancer. The potential to truly transform some types of metastatic oncogene-addicted cancers into chronic diseases may now lie within our reach.

## Abbreviations

ALK: Anaplastic lymphoma kinase
CML: Chronic myeloid leukemia
CRC: Colon and rectum carcinoma
CSF1R: Colonial stimulating factor-1 receptor
DTC: Differential thyroid carcinoma
EGFR: Epidermal growth factor receptor
EML4-ALK: Echinoderm microtubule associated protein-like 4-anaplastic lymphoma kinase
EMT: Epithelial-mesenchymal transition
GIST: Gastrointestinal stromal tumor
HCC: Hepatic cellular carcinoma
HGFR: Hepatocyte growth factor receptor
InsR: Insulin receptor
NRTK: Non-receptor PTK
NSCLC: Non-small cell lung cancer
PDGFR: Platelet-derived growth factor receptor
PTK: Protein tyrosine kinase
RCC: Renal cell carcinoma
RTK: Receptor PTK
SCGFR: Stem cell growth factor receptor
STS: Soft tissue sarcoma
TKI: Tyrosine kinase inhibitor
VEGFR: Vascular endothelial growth factor receptor
